# Retinal degeneration-3 protein attenuates photoreceptor degeneration in transgenic mice expressing dominant mutation of human retinal guanylyl cyclase

**DOI:** 10.1016/j.jbc.2021.101201

**Published:** 2021-09-16

**Authors:** Igor V. Peshenko, Elena V. Olshevskaya, Alexander M. Dizhoor

**Affiliations:** Pennsylvania College of Optometry, Salus University, Elkins Park, Pennsylvania, USA

**Keywords:** cyclic GMP, eye, calcium-binding proteins, GCAP, guanylate cyclase (guanylyl cyclase), GUCY2D, photoreceptor, RetGC, retinal degeneration, RD3, CNG, cyclic nucleotide–gated channel, CORD6, cone–rod dystrophy 6, ERG, electroretinography, GCAP, guanylyl cyclase–activating protein, HEK293, human embryonic kidney cells 293, LCA12, Leber's congenital amaurosis type 12, OCT, optical coherence tomography, ONL, outer nuclear layer, RD3, retinal degeneration-3 protein, RetGC, the retinal membrane guanylyl cyclase, ROS, rod outer segment, TTBS, Tris-buffered saline containing 0.5% Tween-20

## Abstract

Different forms of photoreceptor degeneration cause blindness. Retinal degeneration-3 protein (RD3) deficiency in photoreceptors leads to recessive congenital blindness. We proposed that aberrant activation of the retinal membrane guanylyl cyclase (RetGC) by its calcium-sensor proteins (guanylyl cyclase–activating protein [GCAP]) causes this retinal degeneration and that RD3 protects photoreceptors by preventing such activation. We here present *in vivo* evidence that RD3 protects photoreceptors by suppressing activation of both RetGC1 and RetGC2 isozymes. We further suggested that insufficient inhibition of RetGC by RD3 could contribute to some dominant forms of retinal degeneration. The R838S substitution in RetGC1 that causes autosomal-dominant cone–rod dystrophy 6, not only impedes deceleration of RetGC1 activity by Ca^2+^GCAPs but also elevates this isozyme's resistance to inhibition by RD3. We found that RD3 prolongs the survival of photoreceptors in transgenic mice harboring human R838S RetGC1 (*R838S*^*+*^). Overexpression of GFP-tagged human RD3 did not improve the calcium sensitivity of cGMP production in *R838S*^*+*^ retinas but slowed the progression of retinal blindness and photoreceptor degeneration. Fluorescence of the GFP-tagged RD3 in the retina only partially overlapped with immunofluorescence of RetGC1 or GCAP1, indicating that RD3 separates from the enzyme before the RetGC1:GCAP1 complex is formed in the photoreceptor outer segment. Most importantly, our *in vivo* results indicate that, in addition to the abnormal Ca^2+^ sensitivity of R838S RetGC1 in the outer segment, the mutated RetGC1 becomes resistant to inhibition by RD3 in a different cellular compartment(s) and suggest that RD3 overexpression could be utilized to reduce the severity of cone–rod dystrophy 6 pathology.

Cyclic GMP mediates rod and cone responses to light by regulating cGMP-gated channels in the photoreceptor outer segment, which open in the dark but close when illumination activates rapid cGMP hydrolysis by the rhodopsin–transducin–phosphodiesterase-6 cascade (reviewed in Refs. (1, 2, 3, 4)). Negative Ca^2+^ feedback controls the rate at which the retinal membrane guanylyl cyclase (RetGC) isozymes, RetGC1 (*GUCY2D*) and RetGC2 (*GUCY2F*) (5, 6, 7), produce cGMP in photoreceptor outer segments: when the influx of Ca^2+^ *via* the cyclic nucleotide–gated (CNG) channels stops, the catalytic activity of RetGC becomes accelerated by the Mg^2+^-liganded form of guanylyl cyclase–activating proteins (GCAPs), the Ca^2+^/Mg^2+^ sensor proteins (reviewed in Refs. (1, 4, 8, 9)). Activation of RetGC continues until cGMP reopens the CNG channels during the recovery phase of the response and resumes the influx of Na^+^ and Ca^2+^. Consequently, GCAPs bind Ca^2+^ and decelerate RetGC to prevent excessive production of cGMP and avoid opening more CNG channels. Some mutations in the human *GUCY2D* gene coding for RetGC1 cause inherited dominant retinopathies by deregulating Ca^2+^ sensitivity of RetGC1 (reviewed in Refs. (10, 11, 12)). In particular, various substitutions of Arg^838^ in the RetGC1 dimerization domain frequently cause autosomal dominant cone–rod dystrophy 6 (CORD6) (10, 11, 12). Several *GUCY2D* CORD6-linked mutations (13, 14), including R838S, have been biochemically characterized *in vitro* (13, 14, 15, 16) and *in vivo* (17, 18), demonstrating that in transgenic mice the mutated RetGC1 requires higher free Ca^2+^ concentrations to decelerate the cyclase activity and therefore abnormally elevates cGMP production and Ca^2+^ influx in the dark, leading to progressive photoreceptor degeneration.

Such Arg^838^ substitutions increase RetGC1 affinity for Mg^2+^GCAP, the activator form of GCAP, over the inhibitory form, Ca^2+^GCAP (13, 14, 15, 16). This alters two main biochemical aspects of the guanylyl cyclase regulation in photoreceptors. First, deceleration of the mutated RetGC1 now requires higher than normal free Ca^2+^ concentrations, which in turn elevates cGMP production and the influx of Ca^2+^ (16, 17). The apoptotic changes in photoreceptors triggered by the elevated levels of cGMP and Ca^2+^ in the outer segment can be therefore regarded as a “phototransduction disease” caused by deregulated Ca^2+^ feedback (17). Second, the increased affinity for Mg^2+^GCAP makes the mutated RetGC1 more resistant to inhibition by RD3 protein (16, 18).

RD3 was first identified as the product of a gene disrupted by mutations causing recessive degenerative retinal blindness in humans (Leber's congenital amaurosis type 12 [LCA12]) and in *rd3* mouse strain (*Rd3*^*−/−*^) (19). RD3 is a low-abundance protein required for RetGC to be effectively transferred from the photoreceptor inner segment to the outer segment (20, 21, 22). RD3 also strongly inhibits RetGC basal activity and activation by GCAPs (23, 24). The *in vivo* evidence suggests that aberrant guanylyl cyclase activation by GCAPs in the absence of RD3 provokes the apoptotic process (25, 26), but this does not fully exclude the possibility that GCAPs mediate the apoptotic process *via* some target(s) other than RetGC (27). Therefore, we tested here the effects of deleting RetGC1 or RetGC2 in *Rd3*^*−/−*^ retinas. We reasoned that if GCAPs mediated photoreceptor degeneration independently of RetGC, then the reduction of RetGC content would not affect, or possibly even increase, the progression of photoreceptor loss. Conversely, if the main cause of the *Rd3*^*−/−*^ photoreceptor degeneration were their inability to suppress guanylyl cyclase, then *Rd3*^*−/−*^*RetGC1*^*−/−*^ and/or *Rd3*^*−/−*^*RetGC2*^*−/−*^ photoreceptors would degenerate more slowly than *Rd3*^*−/−*^.

The main hypothesis addressed in this study is that the lower susceptibility of the CORD6 RetGC1 variants to inhibition by RD3 (23, 24) contributes to the progression of this dominant retinal dystrophy. This hypothesis emerged from *in vitro* studies showing that GCAP1-activated R838S RetGC1 requires higher concentrations of RD3 to decelerate its activity. We reasoned that if the increased resistance to inhibition by RD3 aggravates the pathology caused by the Arg^838^ substitution in RetGC1, then increased expression of RD3 could slow the pace of degeneration.

We here present the evidence that (i) the removal of RetGC1 or RetGC2 delays *Rd3*^*−/−*^ photoreceptor degeneration, thus confirming that RD3 protects photoreceptors mainly by suppressing aberrant RetGC activity and (ii) transgenic overexpression of RD3 in mice harboring R838S RetGC1 (*R838S*^*+*^) prolongs the survival of *R838S*^*+*^ photoreceptors. Our findings indicate that RD3 is involved in protecting photoreceptors from two different types of retinal degeneration—the recessive, such as LCA12, and the dominant, such as CORD6.

## Results

### Deletion of guanylyl cyclase isozymes RetGC1 or RetGC2 slows degeneration of *Rd3*^*−/−*^ photoreceptors

**Figure 1:**
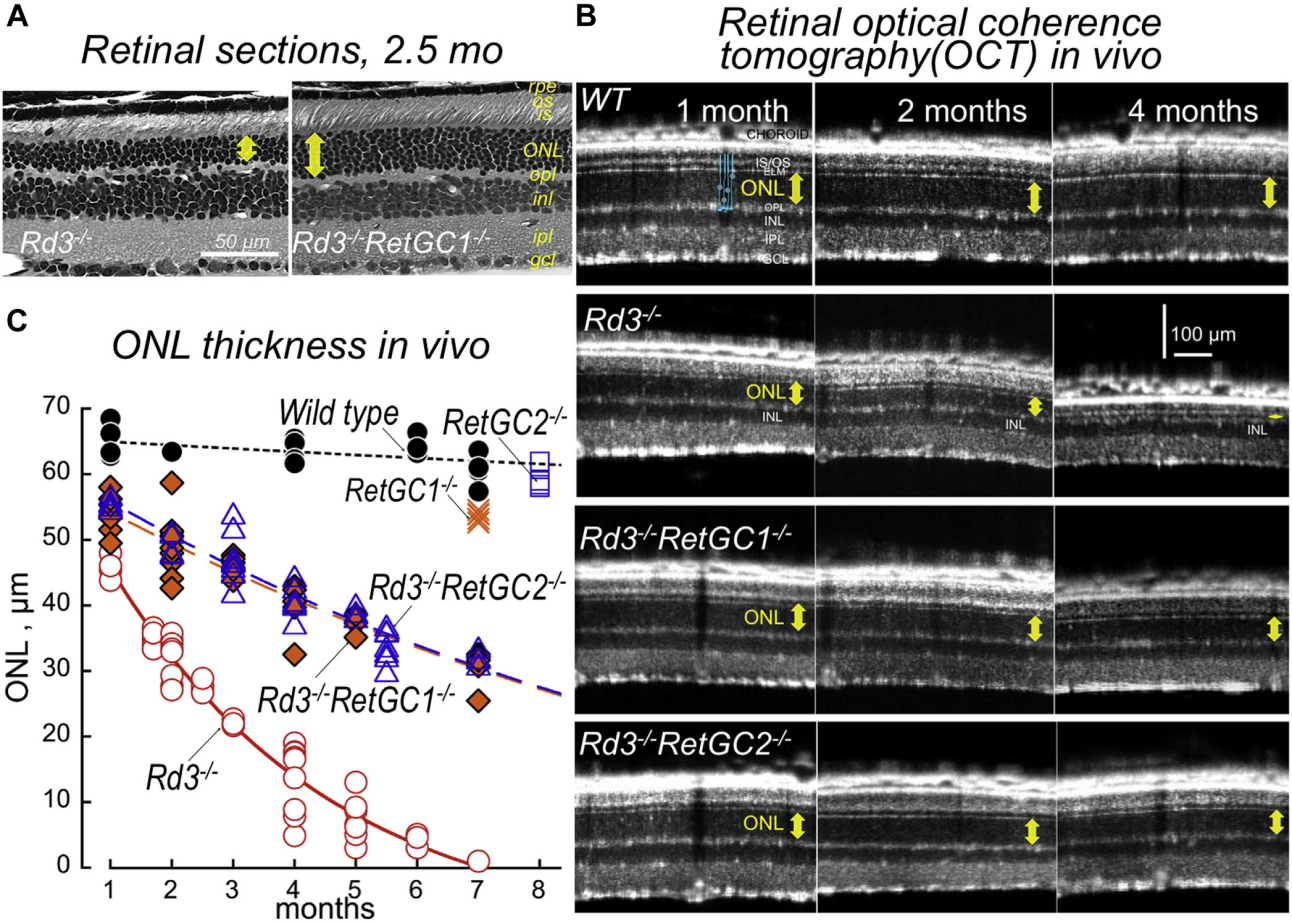
**Deletion of RetGC isozymes slows degeneration of *Rd3***^***−/−***^**photoreceptors.***A*, representative post-mortem retinal morphology in *Rd3*^*−/−*^ (*left*) and *Rd3*^*−/−*^*RetGC1*^*−/−*^ (*right*) littermates at 2.5 months of age. Photoreceptor outer nuclei layer (ONL) thickness hereafter is marked by the *yellow arrow*; other histological layers hereafter are as follows: GCL, ganglion cell layer; INL, inner nuclear layer; IPL, inner plexiform layer; IS, inner segments; OPL, outer plexiform layer; OS, rod outer segments; RPE, retinal pigment epithelium. *B*, representative *in vivo* OCT of the retina at 1, 2, and 4 months of age in (*top* to *bottom*) wildtype, *Rd3*^*−/−*^, *Rd3*^*−/−*^*RetGC1*^*−/−*^, and *Rd3*^*−/−*^*RetGC2*^*−/−*^ mice; note the markedly improved *Rd3*^*−/−*^ ONL thickness after deletion of either RetGC isozyme at 2 and 4 months; the location of photoreceptors is schematically shown in *blue*; bars in either direction—100 μm. *C*, progressing reduction of ONL thickness measured *in vivo* using OCT: wildtype (•), *Rd3*^*−/−*^ (◯), *RetGC1*^*−/−*^ (✕), *RetGC2*^*−/−*^ (□), *Rd3*^*−/−*^*RetGC1*^*−/−*^ (◆), and *Rd3*^*−/−*^*RetGC2*^*−/−*^(△); each data point is an average of ten measurements of ONL in retinal OCT sections per different mouse of indicated genotype; data fitted assuming exponential decay; note the much slower progression of the ONL loss in *Rd3*^*−/−*^ after deletion of RetGC1 or RetGC2. OCT, optical coherence tomography; RetGC, the retinal membrane guanylyl cyclase.

In wildtype (*Rd3*^*+/+*^) mice, simultaneous deletion of both RetGC1 and RetGC2 completely disables cGMP production, eliminates the photoreceptor function, and causes a slowly progressing degeneration of photoreceptors (25, 28). We reasoned that studying the en masse degenerating *RetGC1*^*−/−*^*RetGC2*^*−/−*^ double-knockout photoreceptors lacking RD3 would make it rather cumbersome to isolate the potential contribution of RetGCs to the degeneration caused by the RD3 deficiency *per se*. In contrast, the individual gene knockouts, *RetGC1*^*−/−*^ or *RetGC2*^*−/−*^, only reduce the levels of cGMP synthesis and alter photoresponses but do not entail massive photoreceptor degeneration (28, 29). So the *Rd3*^*−/−*^ degeneration in *RetGC1*^*−/−*^ or *RetGC2*^*−/−*^ background would be caused by RD3 deficiency rather than the RetGC isozymes deficiency, making it possible to assess any potential role of RetGC activity in *Rd3*^*−/−*^ degeneration. We therefore produced *Rd3*^*−/−*^ in either *RetGC1*^*−/−*^ or *RetGC2*^*−/−*^, both congenic with the C57B6 strain background (24). The reduction of the outer nuclear layer (ONL) of photoreceptors assessed by post-mortem morphological analysis and optical coherence tomography (OCT) *in vivo* showed that the rapid loss of the *Rd3*^*−/−*^ ONL was dramatically delayed after deletion of RetGC1 or RetGC2 (Fig. 1, *A* and *B*). In all combinations, including *Rd3*^*−/−*^, the ONL was significantly thinner than in the wildtype (ANOVA, *F* = 184, Scheffé post hoc test *p* < 0.0001, confidence level 99% hereafter), but whereas only 20% normal nuclei count remained after 4 months in *Rd3*^*−/−*^ retinas (Fig. 1, *B* and *C*), the respective 63% and 64% remained in *Rd3*^*−/−*^*RetGC1*^*−/−*^ and *Rd3*^*−/−*^*RetGC2*^*−/−*^, both significantly higher than in *Rd3*^*−/−*^ (*p* < 0.0001). This result conforms to the hypothesis that the primary cause of *Rd3*^*−/−*^ photoreceptor rapid degeneration is the remaining RetGC activity unsuppressed by RD3.

### Overexpression of RD3 does not alter the abnormal regulation of RetGC1 in *R838S*^*+*^ retinas

In order to test the hypothesis that the reduced sensitivity of R838S RetGC1 to inhibition by RD3 contributes to photoreceptor death in *R838S*^*+*^ rods, we overexpressed a human RD3 tagged with GFP at the C terminus in mouse line 362, in which rods transgenically express a human R838S RetGC1 (16, 17) and therefore undergo rapid degeneration. We reasoned that the increase of RD3 concentrations in *R838S*^*+*^ rods could compensate for the reduction of the R838S RetGC1/GCAP1 sensitivity to RD3 (23, 24). To enable monitoring the localization of the expressed RD3, we used RD3GFP that was previously shown to inhibit RetGC activity similarly to untagged RD3 and to fully rescue *Rd3*^*−/−*^ rods from degeneration *in vivo* (25).

**Figure 2:**
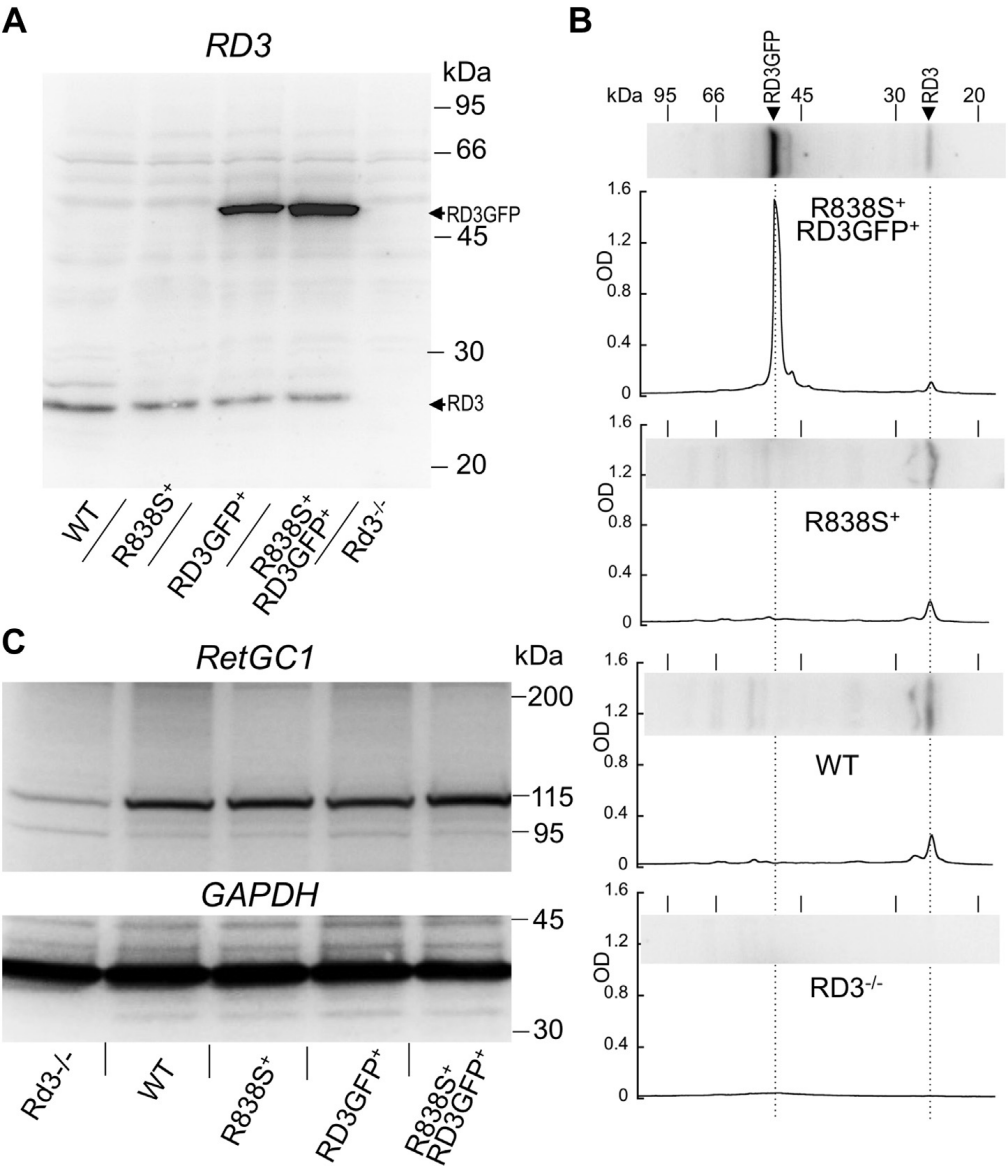
**Overexpression of RD3 in *R838S***^***+***^**retinas.***A* and *B*, immunoblotting of the retinas from indicated genotypes were probed by antimouse RD3 antibody. *B*, the relative intensity of the RD3GFP *versus* the endogenous RD3 images on densitometry scans of the endogenous RD3 and the transgenically expressed human RD3GFP in (*bottom* to *top*) *Rd3*^*−/−*^ (as negative control), wildtype, *R838S*^*+*^, and *R838S*^*+*^*RD3GFP*^*+*^ (all tracks in panel *B* are from the same blot). *C*, immunoblotting from the retinas of indicated genotypes probed by anti-RetGC1 antibody (*top*) or anti-GAPDH (*bottom*) antibody. RD3, retinal degeneration-3 protein.

The overexpression of RD3GFP (Fig. 2) did not significantly affect regulation of the guanylyl cyclase activity in *R838S*^*+*^ retinas. The levels of RetGC expression in *R838S*^*+*^ and *R838S*^*+*^*RD3GFP*^*+*^ were similar, whereas the RD3 content was markedly increased in *R838S*^*+*^*RD3GFP*^*+*^ as compared with wildtype or *R838S*^*+*^. The expressed RD3GFP was of human origin, which could potentially reduce its reactivity toward the antimouse RD3 antibody. But even assuming that its immunoreactivity is similar to that of endogenous mouse RD3 and further taking into account the molecular mass being nearly twice that of the endogenous RD3 (Fig. 2*B*), the total RD3 content, the endogenous plus the transgenically expressed, in *R838S*^*+*^*RD3GFP*^*+*^ was at least fourfold to fivefold higher than normal (possibly even higher if the affinity of the antibody for the human RD3GFP was lower).

**Figure 3:**
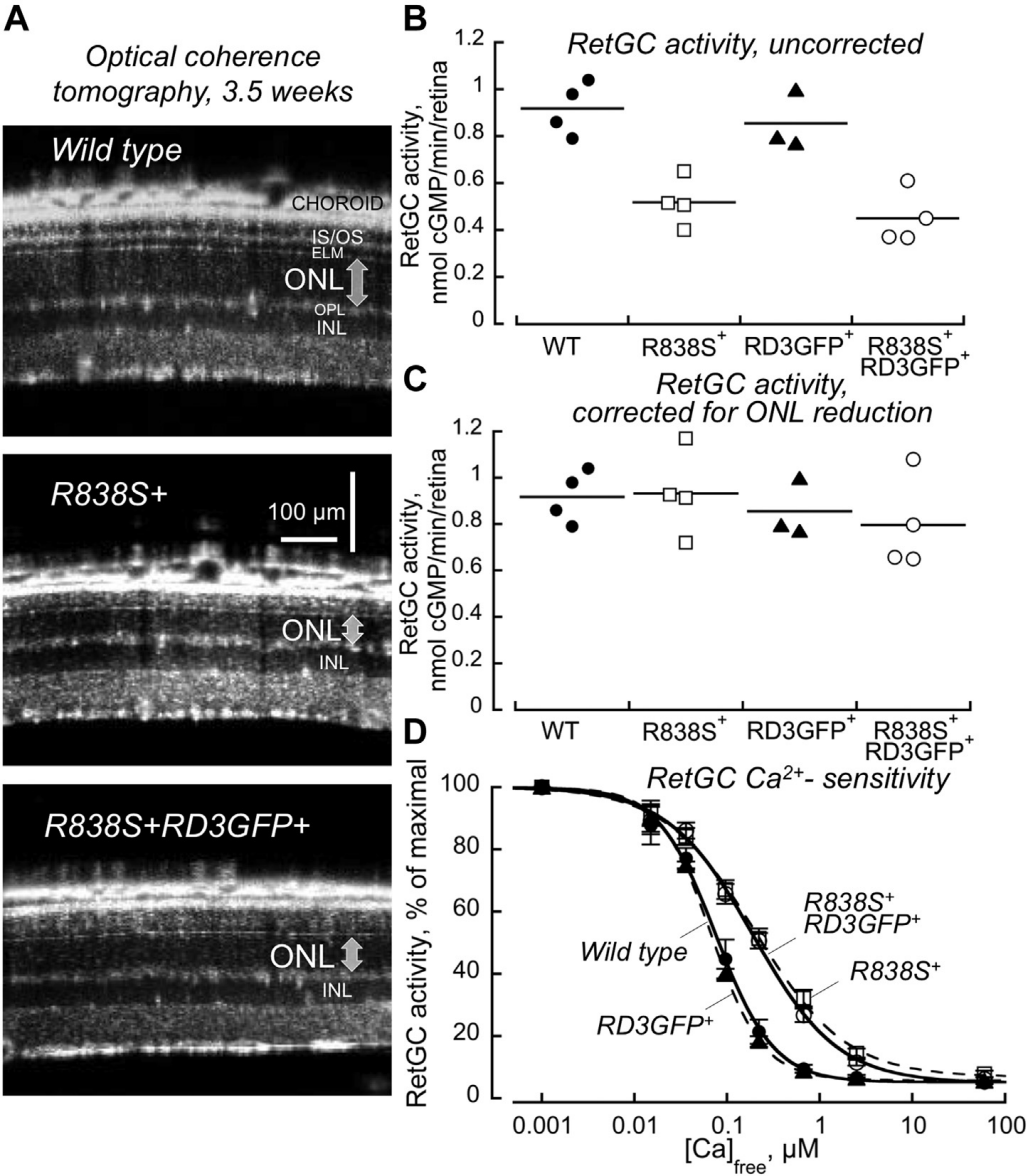
**Overexpression of RD3 does not affect photoreceptor guanylyl cyclase activity in *R838S***^***+***^**retinas.***A*, OCT *in vivo* scans comparing the photoreceptor nuclei mass in wildtype (*top*) degenerating *R838S*^*+*^ and *R838S*^*+*^*RD3GFP*^*+*^ at 3.5 weeks of age; the *arrow* marks photoreceptor ONL thickness. *B* and *C*, RetGC activity measured in the presence of 1 mM EGTA in the retinas from 3.5-week-old mice before (*B*) or after (*C*) correction for the loss of photoreceptor ONL layer: wildtype (•), *R838S*^*+*^(□), *RD3GFP*^*+*^ (▲), and *R838S*^*+*^*RD3GFP*^*+*^(◯). *D*, normalized Ca^2+^ sensitivity of RetGC activity in wildtype (•), *R838S*^*+*^(□), *RD3GFP*^*+*^ (▲), and *R838S*^*+*^*RD3GFP*^*+*^(◯) retinas. See Experimental procedures section for details. OCT, optical coherence tomography; ONL, outer nuclear layer; RD3, retinal degeneration-3 protein; RetGC, the retinal membrane guanylyl cyclase.

Also, the overexpression of RD3 in *R838S*^*+*^ *RD3GFP*^*+*^ did not critically affect overall RetGC activity or its calcium sensitivity (Fig. 3, *A*–*C*). After 3 weeks of age, the early stage of degeneration only partially reducing the photoreceptor content, the bulk activity of the photoreceptor guanylyl cyclase in *R838S*^*+*^ and *R838S*^*+*^*RD3GFP*^*+*^ retinas appeared lower than normal. However, it became similar to the wildtype after being corrected for the partial reduction of the photoreceptor mass as compared with wildtype by measuring the ONL thickness *in vivo* (Fig. 3, *A* and *C*). Overexpression of RD3GFP in wildtype retinas did not significantly alter the normal GCAP-dependent Ca^2+^ sensitivity of the cGMP production ([Ca]_1/2_, mean ± SD): 0.067 ± 0.003 μM *versus* 0.076 ± 0.012 μM (*p* = 0.25, Student's *t* test) (Fig. 3*D*). Notably, the abnormal Ca^2+^ sensitivity of RetGC in *R838S*^*+*^*RD3GFP*^*+*^ retinas also remained identical to its abnormal Ca^2+^ sensitivity in *R838S*^*+*^ (Fig. 3*D*). The [Ca]_1/2_ for deceleration of RetGC activity, 0.191 ± 0.021 and 0.203 ± 0.026 μM, respectively, was both markedly different from the wildtype (*p* < 0.0008) but virtually indistinguishable from each other (*p* = 0.50).

### Overexpression of RD3 delays degeneration of *R838S*^*+*^ rods

**Figure 4:**
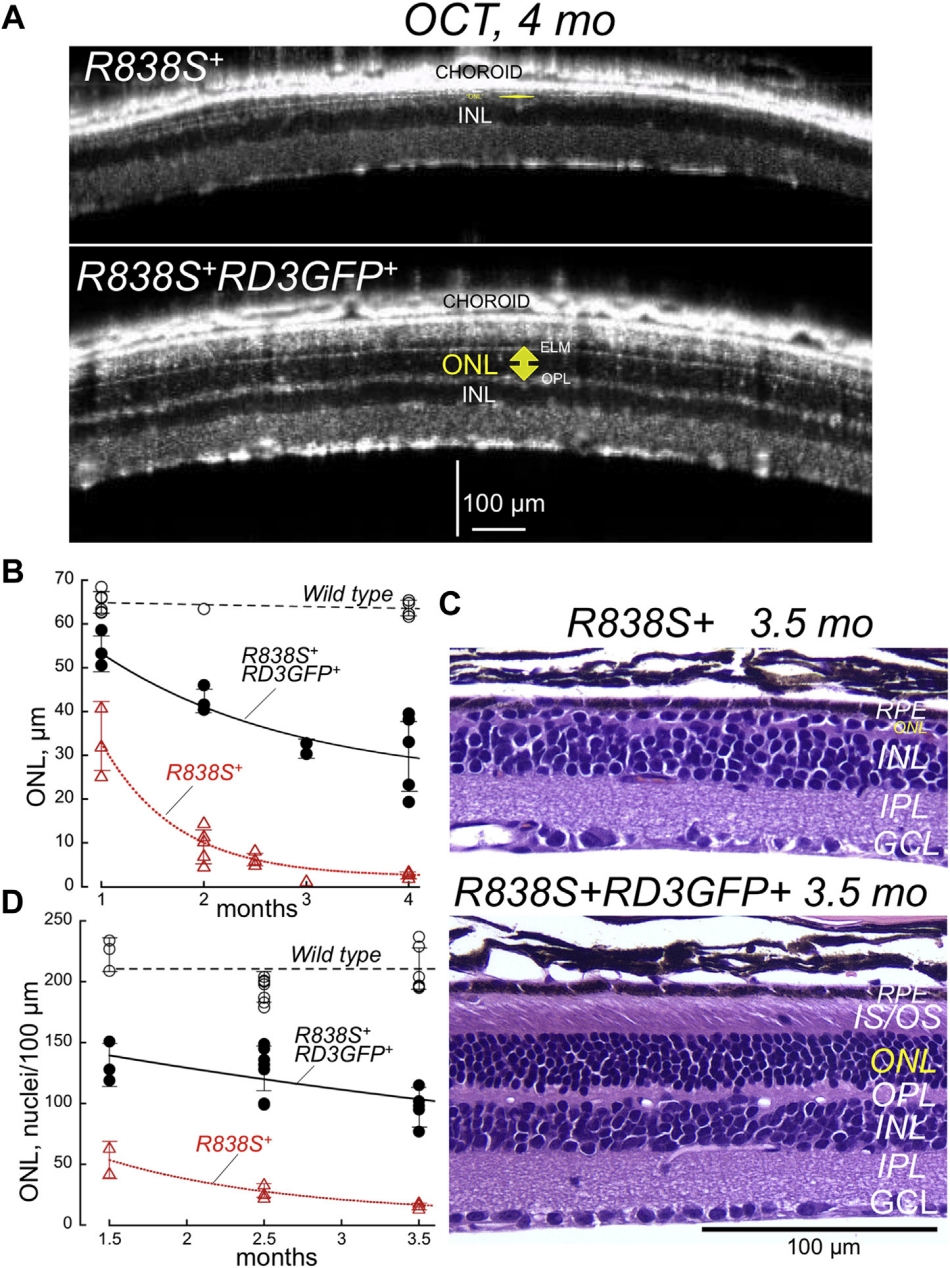
**RD3 overexpression partially rescues *Rd3***^***−/−***^**rod degeneration.***A*, representative retinal OCT images of *R838S*^*+*^ and *R838S*^*+*^*RD3GFP*^*+*^ littermates at 4 months of age; note the complete lack of photoreceptors in *R838S*^*+*^ and the preservation of identifiable ONL in *R838S*^*+*^*RD3GFP*^*+*^. *B*, quantitation of the ONL in wildtype (◯), *R838S*^*+*^ (△), and *R838S*^*+*^*RD3GFP*^*+*^ (•) by OCT *in vivo* at different ages; error bars represent SD. *C*, representative post-mortem retinal morphology in histological sections from *R838S*^*+*^ and *R838S*^*+*^*RD3GFP*^*+*^ littermates at 3.5 months of age; note the nearly complete lack of identifiable photoreceptor nuclei in *R838S*^*+*^ and their abundant presence in *R838S*^*+*^*RD3GFP*^*+*^ along with the identifiable histological layer of rod inner and outer segments. *D*, linear density of photoreceptor nuclei in wildtype (◯), *R838S*^*+*^ (△), and *R838S*^*+*^*RD3GFP*^*+*^ (•) counted using histological sections. OCT, optical coherence tomography; ONL, outer nuclear layer; RD3, retinal degeneration-3 protein.

Both *R838S*^*+*^ and *R838S*^*+*^*RD3GFP*^*+*^ developed photoreceptor degeneration. Yet, despite retaining the same abnormal Ca^2+^ sensitivity of RetGC as in their parental *R838S*^*+*^ line, the degenerative changes of retinal morphology in *R838S*^*+*^*RD3GFP*^*+*^ mice developed much more slowly than in *R838S*^*+*^ siblings (Fig. 4). The *in vivo* OCT scans revealed markedly improved ONL thickness after 4 months (31 ± 9 *versus* 3 ± 1 μm in *R838S*^*+*^; Student’s *t* test, *p* = 0.0023) (Fig. 4*A*). In contrast to *R838S*^*+*^, where the photoreceptor layer completely degenerated within 3.5 months, the ONL thickness in *R838S*^*+*^*RD3GFP*^*+*^ gradually declined to 50% wildtype at the end of the 4-month period (Fig. 4*B*). Likewise, in a post-mortem histological analysis of the retinas (Fig. 4, *C* and *D*), photoreceptor nuclei count per 100 μm in the *R838S*^*+*^*RD3GFP*^*+*^ retinas was lower than in wildtype (97 ± 14, n = 3 *versus* 210 ± 17, n = 6; *p* < 0.0001), yet dramatically improved compared with *R838S*^+^ (17 ± 3, n = 5; *p* = 0.0001). The remaining 49% of the photoreceptors in *R838S*^*+*^*RD3GFP*^*+*^ retained recognizable outer segment layer, whereas no recognizable photoreceptor structures remained in *R838S*^+^ (Fig. 4*D*).

### RD3GFP overexpression delays the loss of electroretinography responses in *R838S*^*+*^ retinas

**Figure 5:**
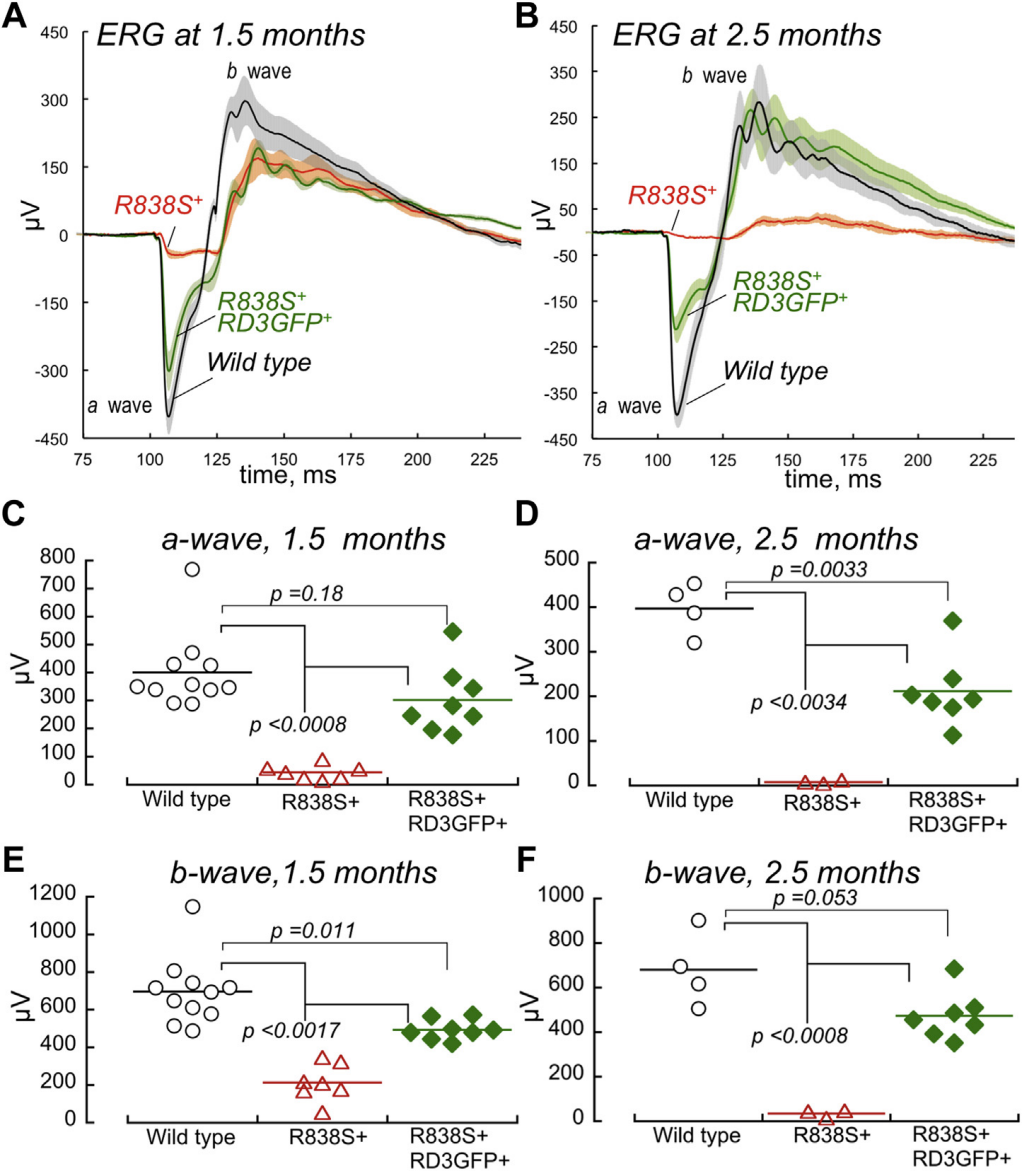
**RD3 overexpression offsets the loss of rod function in *R838S***^***+***^**.***A* and *B*, dark-adapted ERG (mean ± SEM error bars) recorded in wildtype (*black traces*, *gray error bars*), *R838S*^*+*^ (*red traces*, *pale orange error bars*), and *R838S*^*+*^*RD3GFP*^*+*^ (*green traces*, *pale green error bars*) mice at 1.5 months (*A*) and 2.5 months (*B*) of age in response to a 1-ms 505-nm flash of ∼1 × 10^6^ photons/rod delivered at 100 ms on the timescale. *C* and *D*, the a-wave amplitudes in wildtype (◯), *R838S*^*+*^ (△), and *R838S*^*+*^*RD3GFP*^*+*^ (◆) mice at 1.5 months of age (*C*) and 2.5 months of age (*D*). *E* and *F*, the respective b-wave amplitudes at 1.5 months of age (*E*) and 2.5 months of age (*F*). ERG, electroretinography; RD3, retinal degeneration-3 protein.

Retinal function assessed by dark-adapted electroretinography (ERG) was also preserved in *R838S*^*+*^*RD3GFP*^*+*^ much better than in *R838S*^*+*^ (Fig. 5). The ERG a-wave (negative voltage deflection produced by hyperpolarization of photoreceptors in response to a bright flash) was strongly suppressed at 1.5 months of age (44 ± 26 μV; mean ± SD) (Fig. 5*A*) and virtually undetectable (<10 μV) at 2.5 months of age in *R838S*^*+*^ (Fig. 5*B*) because of rapidly progressing *en masse* loss of photoreceptors. In comparison with *R838S*^*+*^, the ERG a-wave amplitudes in *R838S*^*+*^*RD3GFP*^*+*^ were much larger both at 1.5 months (302 ± 120 μV, *p* = 0.0008) (Fig. 5, *A* and *C*) and at 2.5 months (212 ± 79 μV; *p* < 0.0001) (Fig. 5, *B* and *D*) of age. They were only slightly lower in *R838S*^*+*^*RD3GFP*^*+*^ than the wildtype (401 ± 135 μV; *p* = 0.18) at 1.5 months (Fig. 5, *A* and *C*) but more significantly (*p* = 0.0033) reduced (212 ± 79 μV) than the 397 ± 58 μV in the wildtype at 2.5 months (Fig. 5*B*), as *R838S*^*+*^*RD3GFP*^*+*^ photoreceptors continued to gradually degenerate, albeit much slower than in *R838S*^*+*^. The b-wave generated by inner retina neurons receiving input from photoreceptors (positive voltage deflection measured from the bottom of the a-wave), despite being lower than in wildtype, was also drastically improved in *R838S*^*+*^*RD3GFP*^*+*^ as compared with *R838S*^*+*^, both at 1.5 (Fig. 5*E*) and 2.5 months (Fig. 5*F*).

### RD3 is displaced from the complex with RetGC1 in the outer segment

**Figure 6:**
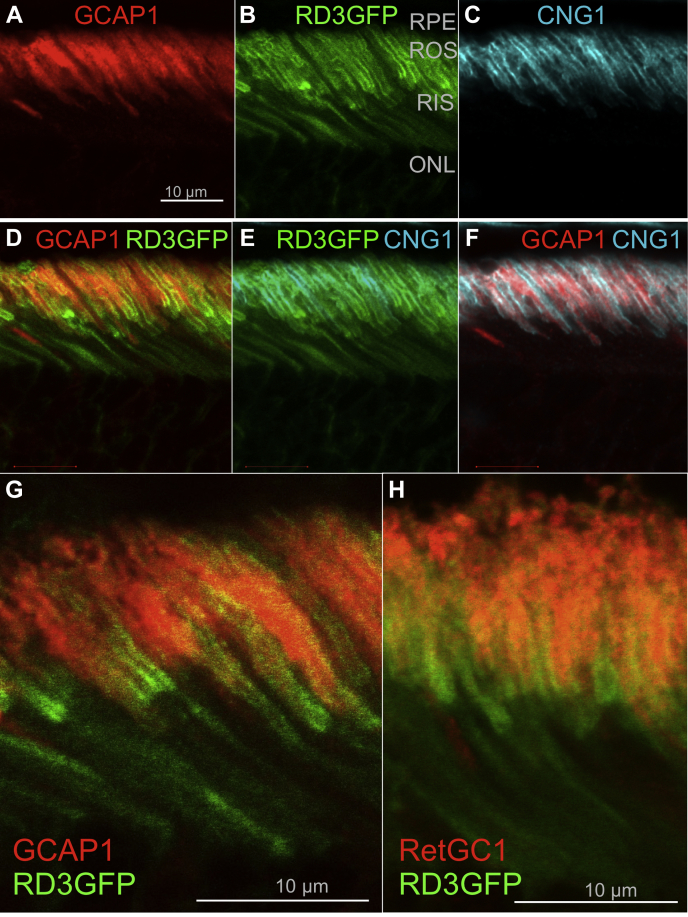
**RD3GFP separates from the RetGC1 and GCAP1 accumulated in ROS.***A*–*F*, immunofluorescence in 3.5-week-old *RD3GFP*^*+*^ retinas probed with anti-GCAP1 rabbit polyclonal antibody (*red*) (*A*, *D*, and *F*) or anti-CNG1 mouse polyclonal antibody (pseudocolored *cyan*; *C*, *E*, and *F*) in comparison with RD3GFP fluorescence (*green*; *B*, *D*, *E*, and *F*); the bar represents 10 μm. *G* and *H*, separation of RD3GFP from GCAP1 (*G*) and RetGC1 (*H*) immunofluorescence in *R838S*^*+*^*RD3GFP*^*+*^ ROS. The *R838S*^*+*^*RD3GFP*^*+*^ retina sections were probed by rabbit polyclonal anti-GCAP1 (*red*, *G*) or RetGC1 (*red*, *H*) antibody. Note the similarity between the GCAP1 and RetGC1 localization inside ROS, different in the both cases from the localization of *green* RD3GFP fluorescence, which is concentrated in the inner segment and near the base of the ROS, partially extending along the plasma membrane but not to the inside of the ROS. CNG, cyclic nucleotide–gated; GCAP, guanylyl cyclase–activating protein; RD3, retinal degeneration-3 protein; RetGC, the retinal membrane guanylyl cyclase; ROS, rod outer segment.

RD3 and GCAP regulate RetGC activity in opposite directions: GCAP activates RetGC, and RD3 inhibits it in a manner indicating that it disables the guanylyl cyclase stimulation by competing with GCAP (23, 24, 25). Conversely, GCAP would have to take over the RetGC regulation in the outer segment, otherwise Ca^2+^ feedback on the cyclase would be disabled (23). Therefore, we tested RD3GFP *versus* GCAP1 and RetGC1 localization in *R838S*^*+*^ and *R838S*^*+*^*RD3GFP*^+^ retinas (Fig. 6).

**Figure 7:**
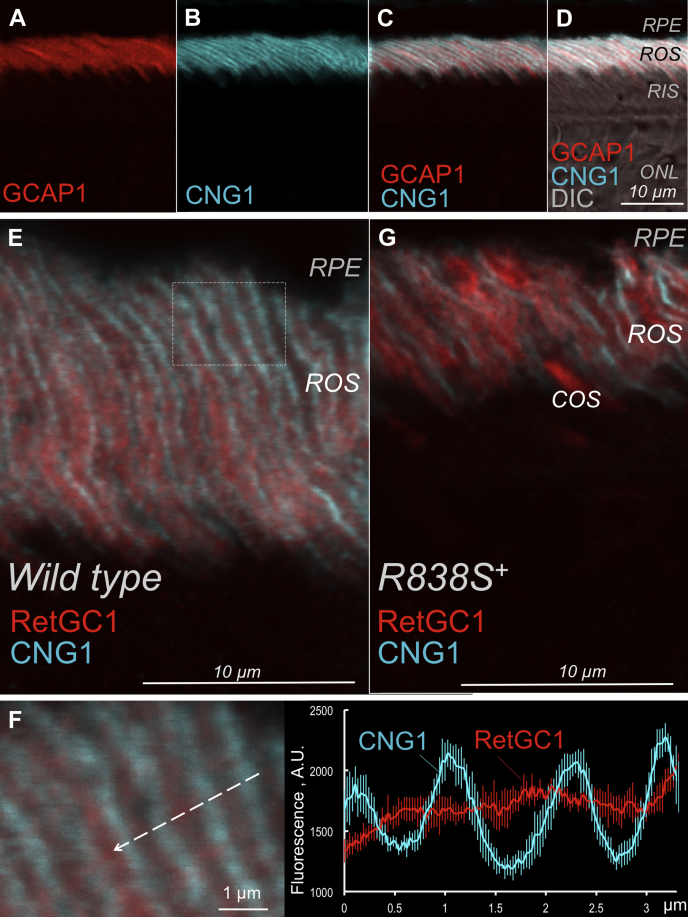
**GCAP1 accumulates with RetGC inside wildtype and *R838S***^***+***^**ROS.***A*–*D*, immunofluorescence in the wildtype retinas probed with anti-GCAP1 rabbit polyclonal antibody (*red*) (*A*, *C*, and *D*) and anti-CNG1 mouse polyclonal antibody (*cyan*) (*B*, *C*, and *D*); the merged anti-GCAP1 and anti-CNG1 fluorescence (*C* and *D*) were superimposed on DIC image (*D*). *E*–*G*, anti-RetGC1 (AlexaFluor 543, *red*) and anti-CNG1 (AlexaFluor 647, pseudocolored *cyan*) immunofluorescence in photoreceptors of 3.5-week-old wildtype (*E* and *F*) and *R838S*^*+*^ (*G*) mice. In *F* (*left panel*), the magnified region of the wildtype retina (marked with the *rectangle* in *E*) was scanned along the *dashed line*; the distribution of the respective fluorescence intensities is shown in the *right panel*. Pearson's correlation coefficient for the two fluorochromes in (*E*) was 0.31 ± 0.25 (n = 8). See Experimental procedures section for details. CNG, cyclic nucleotide–gated; DIC, differential interference contrast image; GCAP, guanylyl cyclase–activating protein; RetGC, the retinal membrane guanylyl cyclase; ROS, rod outer segment.

Whereas the anti-CNG1α fluorescence exclusively marking the rod outer segment (ROS) plasma membrane was distributed along the entire ROS length, RD3GFP fluorescence only partially overlapped with the anti-CNG1α in both *RD3GFP*^+^ and *R838S*^*+*^*RD3GFP*^+^ (Figs. 6 and 7). The bulk of the green RD3GFP fluorescence was observed in proximity to the lower portion of the ROS only partially spreading toward the ROS plasma membrane (Fig. 6): the brightest fluorescence accumulated near the base of the outer segment and was also spread in the inner segment/myoid part of rods (Fig. 6). RetGC1 and GCAP1 immunofluorescence signals (Figs. 6 and 7) did not overlap with CNG1α either (Pearson's correlation coefficient <0.5), but in contrast to RD3GFP, they were both detectable inside *R838S*^*+*^, *R838S*^*+*^*RD3GFP*^*+*^, and wildtype ROS (Fig. 7, *E* and *F*), showing a pattern more consistent with the predominant localization of the cyclase in photoreceptor discs (30) rather than the ROS plasma membrane.

**Figure 8:**
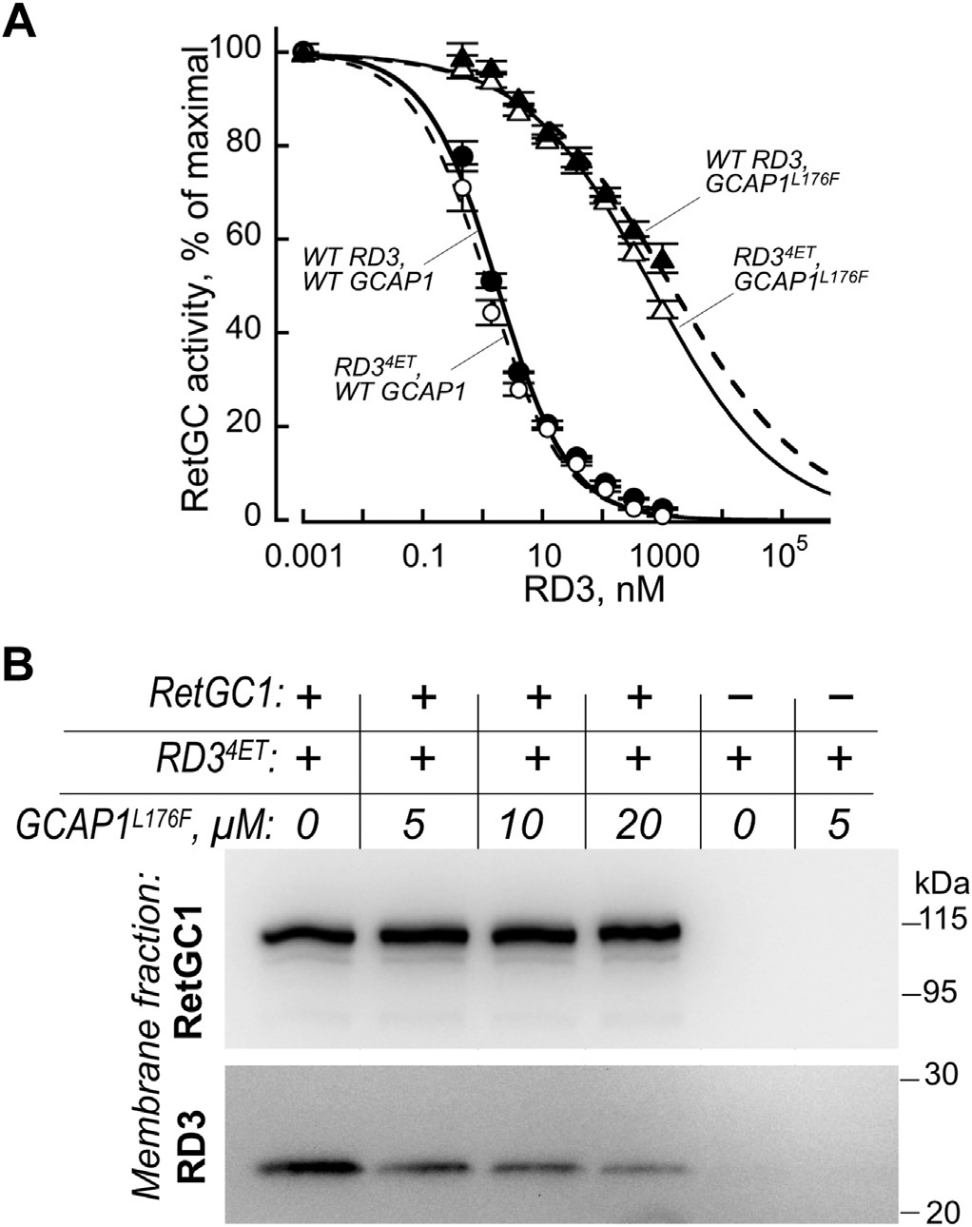
**GCAP can displace RD3 from RetGC1.***A*, inhibition of RetGC1 activity by RD3 *in vitro*. The RetGC1 activity in HEK293 membranes activated by 1.5 μM wildtype Mg^2+^ GCAP1 (●, ◯) or Mg^2+^ GCAP1^L176F^ (▲, △) was assayed in the presence of increasing concentrations of wildtype RD3 (•, ▲) or RD3^4ET^ (◯, △). *B*, cosedimentation assay of RD3^4ET^ binding to RetGC1 using ultracentrifugation. Immunoblots of HEK293 membrane pellets, either containing (+) or not containing (−) expressed RetGC1, were obtained by ultracentrifugation after preincubation with 50 nM RD3^4ET^ in the presence of indicated concentrations of L176F GCAP1. The immunoblot was probed by anti-RetGC1 (*top*) or anti-RD3 (*bottom*) antibody as described in the Experimental procedures section. GCAP, guanylyl cyclase–activating protein; HEK293, human embryonic kidney 293 cells; RD3, retinal degeneration-3 protein; RetGC, the retinal membrane guanylyl cyclase.

We hypothesized that the different compartmentalizations of GCAP and RD3 in photoreceptors result from displacement of RD3 by GCAP from the complex with RetGC, presumably before the guanylyl cyclase reaches its destination in the ROS photoreceptor discs. However, the content of RD3 in the retina is too low (20) to allow for a reliable direct assessment of its complex with the RetGC and possible RD3 displacement by GCAPs. Therefore, to assess whether such displacement is mechanistically possible, we used reconstitution of the recombinant RD3 with RetGC1 expressed in human embryonic kidney 293 (HEK293) cells membranes and tested their binding using cosedimentation after ultracentrifugation (Fig. 8). We found that the micromolar affinity of the RetGC isozymes for GCAPs (Ref. (31) and Fig. 8*A*) is insufficiently strong to allow a stable GCAP1:RetGC1 membrane complex to be isolated in this type of experiment. In contrast, the apparent RetGC affinity for RD3 is at least 100-fold higher, and the RD3:RetGC complex is more stable (20, 23, 24). However, the very poor solubility of RD3 heterologously expressed in bacterial and human cells (23, 32, 33) presents a major challenge because precipitated RD3 nonspecifically contaminates the fraction of the RetGC1-containing membranes. To overcome that obstacle, we produced an RD3 variant (RD3^4ET^) in which Glu replaced Arg in positions 154, 156, 158, and 167, and the last 18 C-terminal residues were deleted. RD3^4ET^ fully retained the inhibitory activity of wildtype RD3 *in vitro* (Fig. 8*A*), but, unlike wildtype RD3, remained in a soluble fraction after ultracentrifugation. The much lower apparent affinity of GCAP1 for RetGC1 than in RD3 presents another major challenge for the binding experiments, requiring unrealistically high concentrations of GCAP1 in the assay. To mitigate this, we used L176F GCAP1, mutation that increases RetGC1 affinity for GCAP1 approximately fivefold (34, 35).

Confirming the specificity of the binding of RD3s with the RetGC1, in the centrifugation assay, the soluble RD3^4ET^ cosedimented with the HEK293 membranes containing RetGC1 but not with HEK293 membranes lacking the expressed guanylyl cyclase (Fig. 8*B*). There was clear evidence that RD3 was bound to the RetGC1-containing membranes in the absence of GCAP1, yet it was displaced from the membrane complex with RetGC1 in a dose-dependent manner in the presence of high concentrations of GCAP1.

## Discussion

### Two RetGC isozymes mediate *Rd3*^*−/−*^ photoreceptor degeneration

**Figure 9:**
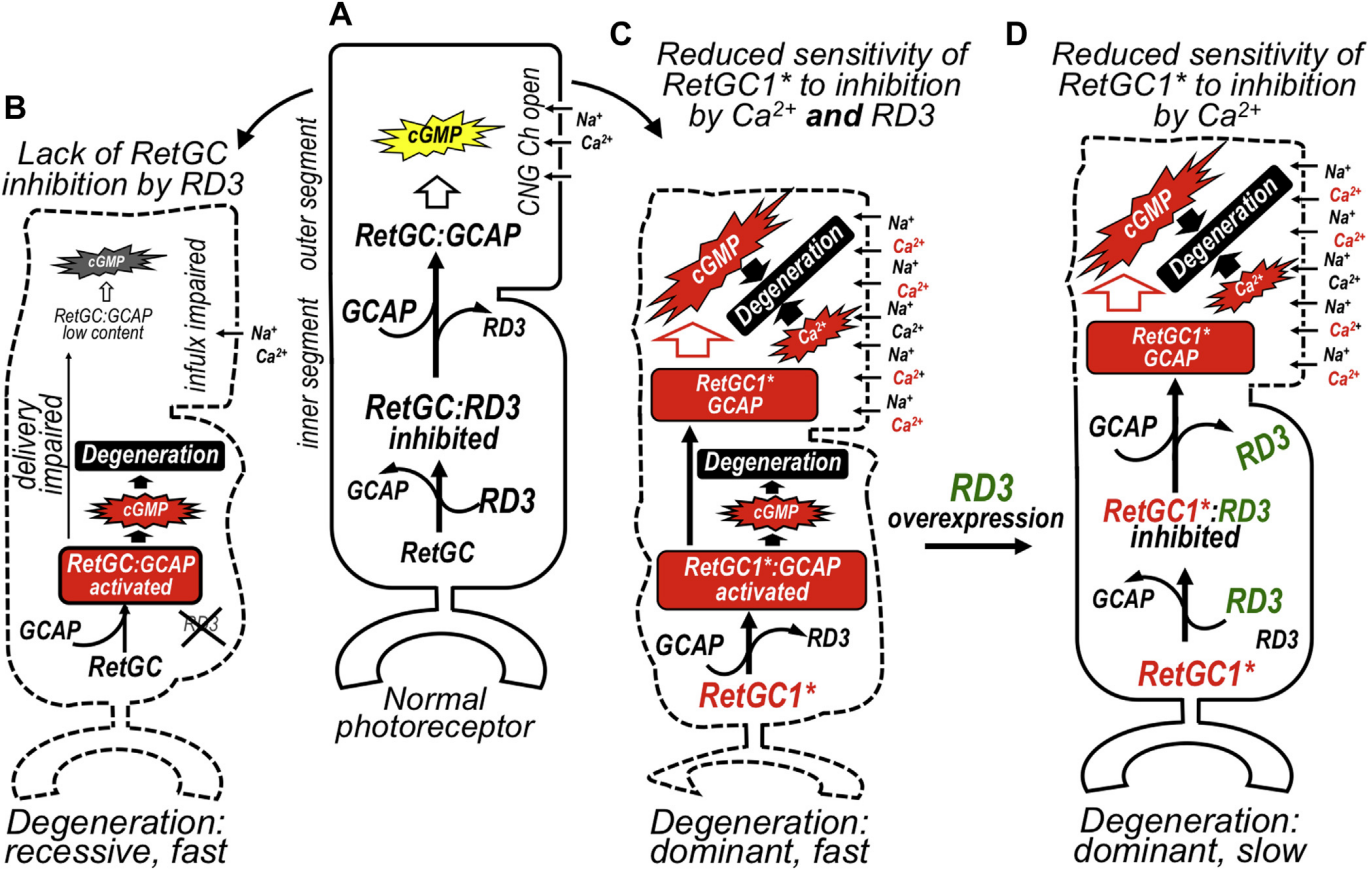
**The putative role of RD3 in preventing two different types of rapid photoreceptor degeneration.***A*, in normal photoreceptors, RD3 plays a dual role: (i) it facilitates RetGC delivery to the outer segment and (ii) protects the cells against aberrant activation of guanylyl cyclase in the inner segment until the enzyme reaches its destination in the outer segment, where GCAPs displace RD3 and control RetGC activity *via* Ca^2+^ feedback. *B*, in RD3-deficient photoreceptors such as in *rd3* mice and LCA12 patients, the RetGC content sharply declines and the production of cGMP in the outer segment becomes insufficient to maintain the normal inward current through CNG channels, which reduces responses to light and possibly provokes a slow component of the degenerative process. The remaining RetGC is no longer protected against aberrant activation by GCAPs, and such activation causes rapid degeneration of the RD3-deficient photoreceptors. *C*, in photoreceptors expressing *GUCY2D* dominant gain-of-function mutations (RetGC1^∗^), such as CORD6-linked mutation R838S, the higher affinity of the RetGC1^∗^ for GCAP causes a “phototransduction disease” by deregulating the suppression of the RetGC1∗ activity by Ca^2+^ in the outer segment, which in turn causes excessive cGMP production and abnormal elevation of Ca^2+^ influx in the dark, promoting the apoptotic process. The increased affinity of RetGC1^∗^ for GCAP also hinders RetGC1^∗^ silencing by RD3. The aberrant stimulation of the cyclase, possibly in the inner segment, activates the secondary pathway of degeneration, similarly to that in RD3-deficient photoreceptors. *D*, the increase of RD3 levels by overexpressing RD3GFP enables RD3 to compete with GCAPs for RetGC1^∗^ more effectively and offsets the secondary, RD3-related, pathway of the apoptosis. Therefore, the dominant photoreceptor degeneration driven now only by the primary apoptotic pathway of deregulated Ca^2+^ feedback in *R838S*^*+*^ outer segment becomes slower. CNG, cyclic nucleotide–gated; CORD6, cone–rod dystrophy 6; GCAP, guanylyl cyclase–activating protein; RD3, retinal degeneration-3 protein; RetGC, the retinal membrane guanylyl cyclase.

RD3 inhibits the RetGC1 and RetGC2 basal activity and also prevents RetGC activation by GCAPs (23, 24) (Fig. 9*A*). Studying murine models *in vivo* (24, 25) indicated that activation of RetGC by GCAPs, most likely in the inner segment, was the major reason that *Rd3*^*−/−*^ photoreceptors begin to rapidly degenerate soon after completing their differentiation (36) (Fig. 9*B*). Deletion of the two GCAP isoforms dramatically rescued *Rd3*^*−/−*^ photoreceptors from degeneration (25, 26, 27). Yet the possibility that the apoptotic effect of GCAPs in *Rd3*^*−/−*^ photoreceptors was propagated not *via* aberrant activation of the guanylyl cyclase but through some other process (27) could not be fully excluded. The most interesting alternative possibility that has been suggested is that GCAP2 causes photoreceptor death by activating in the inner segment an apoptotic pathway(s) in a way that is unrelated to RetGC regulation (27). In our recent study (26), we observed that deletion of GCAP2 indeed led to partial rescue, but it was less effective than deletion of GCAP1 and especially less effective than simultaneous deletion of both GCAPs. The results of our present study demonstrate that removal of either RetGC isozyme markedly reduces the rate of *Rd3*^*−/−*^ photoreceptor degeneration (Fig. 1), similarly to the previously documented deletion of GCAPs (25, 26, 27). Taken together, these results do not support the hypothesis that GCAPs in the absence of RD3 facilitate apoptosis primarily through a process unrelated to activation of RetGC. Instead, the data support the hypothesis that RD3 protects photoreceptors by suppressing aberrant RetGC1 and RetGC2 activities stimulated by GCAPs (Fig. 9*A*) and that the lack of such suppression is the primary cause of *Rd3*^*−/−*^ photoreceptor death.

The strongly defined protective effect of deleting RetGC2 was somewhat surprising. Based on the biochemical evidence, RetGC2 is mostly an ancillary isozyme in mouse rods, accounting for ∼30% of the total guanylyl cyclase activity (31). Therefore, one may expect that RetGC2, which is regulated *in vivo* almost exclusively by GCAP2 (37), would contribute less to the progression of *Rd3*^*−/−*^ photoreceptor death than RetGC1. However, the preservation of *Rd3*^*−/−*^ photoreceptors after deletion of RetGC2 was similar to that of RetGC1 (Fig. 1, *B* and *C*). One possible reason is that RetGC2 in mouse photoreceptors has a higher basal activity than RetGC1 (31). Consequently, the contribution of RetGC2 to the apoptotic process triggered by RD3 deficiency can be higher than its contribution to the total GCAP-stimulated RetGC activity.

Another component causing major dysfunction of *Rd3*^*−/−*^ photoreceptors is the severe reduction of RetGC content (20, 21, 25), resulting in suppression of their photoresponses even before the en masse degeneration occurs (Fig. 5 and Refs. (19, 24, 25)). Even though the low guanylyl cyclase activity *per se* is not the main reason for the rapidly progressing *Rd3*^*−/−*^ degeneration (24), mouse photoreceptors completely lacking RetGC activity are known to slowly degenerate (24, 28). Hence, the decrease in RetGC activity could conceivably facilitate apoptosis *via* drastically lowering cGMP and Ca^2+^ levels in the *Rd3*^*−/−*^ outer segment. More definitive evaluation of such a possibility will require deeper insight into the as-yet insufficiently understood role of RD3 in facilitating RetGC delivery to the outer segment.

### The protective function of RD3 in two different types of photoreceptor degeneration

Deregulation of RetGC1 makes *GUCY2D* CORD6 primarily a “phototransduction disease,” altering the negative Ca^2+^ feedback on RetGC (17). Various mutations linked to CORD6 cause substitutions or Arg^838^ in a human RetGC1 dimerization domain (10, 11, 12, 13, 14). The dimerization domain of RetGC1 is an important structural part of the cyclase enzyme regulated by GCAPs (32, 38). The coiled–coil interactions altered by the Arg^838^ substitutions elevate the affinity of RetGC1 for Mg^2+^GCAP1 (13, 14, 15, 18). As a result, higher concentrations of Ca^2+^ are needed to drive RetGC1 into a complex with Ca^2+^GCAP1 in order to decelerate the cyclase activity so that the normal levels of Ca^2+^ in photoreceptors become insufficient for properly suppressing cGMP production in the dark (16, 17).

The two main apoptotic triggers are considered to be the increased rate of cGMP production in the outer segment and the elevated influx of Ca^2+^ in the dark *via* cGMP-gated CNG channels (39, 40). Both changes occur in the *R838S*^*+*^ mouse rods *in vivo* (17). However, in a previous study (16, 26), we observed that another, more recently recognized aspect of the RetGC regulation—its inhibition by RD3—may contribute to the disease (Fig. 3). The higher affinity of the mutated cyclase for GCAP also makes it more difficult for RD3 to counteract the cyclase activation. Hence, the normal concentrations of RD3 in CORD6 photoreceptors may become insufficient to prevent the aberrant RetGC1 activation by GCAP in the inner segment and hence aggravate the apoptotic process (Fig. 9*C*). If this hypothesis were correct, then one would expect that increasing concentrations of RD3 would reduce the pace of degeneration in an animal model harboring a human RetGC1 with a CORD6 mutation, and in fact, this protective effect of RD3 overexpression in *R8383S*^*+*^ rods was evident in the present study (Figs. 4 and 5).

It is also important to note that despite being evident, the rescue of *R838S*^*+*^*RD3GFP*^*+*^ photoreceptors was not complete and the photoreceptors continued to degenerate, albeit at a much slower pace than in *R838S*^*+*^ littermates. This indicates that the two components in the *R838S*^*+*^ degeneration (Fig. 9*C*) propagate the apoptotic process *via* different pathways—one triggers the degenerative “phototransduction disease” by deregulating Ca^2+^ feedback in the outer segment, and the other by unprotecting the mutant RetGC1 against aberrant activation by GCAPs, either in the inner segment or in a “wrong” part of the outer segment before RetGC1 compartmentalizes with GCAP (Figs. 6 and 7).

The results of our present study (Fig. 8) are consistent with kinetic data (23, 24) indicating that GCAPs displace RD3 from RetGC to enable the Ca^2+^-sensitive regulation of the enzyme in the outer segment. However, it evidently requires very high local concentrations of GCAP to displace RD3 from the complex with the cyclase (Fig. 8), despite the higher than normal affinity for RetGC1 of L176F GCAP1 used in this experiment. It therefore remains unclear how such displacement occurs upon transition of the enzyme from the inner segment to the photoreceptor disc membrane. It is possible that the RetGC1:RD3 complex moves from the inner segment through a compartment containing a high local concentration of GCAP, but it is also possible that RD3, RetGC, and/or GCAP upon entering the outer segment undergo modification(s) that reduce(s) RetGC affinity for RD3 and increases its affinity for GCAP1. These possibilities remain to be explored in future studies.

Expression of RD3 (41) or RD3GFP (25) in *Rd3*^*−/−*^ photoreceptors restores their function and prevents degeneration in a mouse *rd3* model of the human recessive LCA12. Importantly, the results of our present study also indicate that overexpression of RD3 should be further explored as a possible approach to support the survival of photoreceptors expressing human *GUCY2D* with Arg^838^ substitutions. Furthermore, some other types of dominant retinal degeneration originate from mutations in a human *GUCA1A* gene coding for GCAP1 (reviewed in Refs. (9, 12)), and in many cases, such mutations deregulate Ca^2+^ sensitivity of guanylyl cyclase in a manner similar to the Arg^838^ mutations in RetGC1. In at least some of these cases, the affinity of Mg^2+^GCAP1 for RetGC1 can increase and sensitivity to inhibition by RD3 can decrease (42). Additional studies could help shed light on whether degeneration of photoreceptors caused by the dominant mutations in GCAP1 can be partially rescued by overexpression of RD3.

## Experimental procedures

### Animals

All experiments involving animals were conducted in accordance with the Public Health Service guidelines and approved by the Salus University Institutional Animal Care and Use Committee. The wildtype C57B6J and *rd3/rd3* mouse strains were purchased from JAX Research/Jackson's Laboratory. *R838S*^*+*^ mice (line 362) and mice overexpressing RD3GFP (line 932) were produced as described previously (16, 25). Mice of other parental genotypes used in this study were kindly provided by other investigators: *RetGC1*^*−/−*^ line (29)—by Dr David Garbers (University of Texas), and *RetGC2*^*−/−*^ (28)—by Dr Wolfgang Baehr (University of Utah). All mouse lines used in this study were made congenic to the C57B6 background by repetitive breeding for over ten generations prior to conducting the experiments (24). The *RetGC1*^*−/−*^ or *RetGC2*^*−/−*^ mice were bred for several generations to *Rd3*^*−/−*^ in C57B6 background to first produce *Rd3*^*−/−*^*RetGC1*^*+/−*^ or *Rd3*^*−/−*^*RetGC2*^*+/−*^, then the *Rd3*^*−/−*^*RetGC1*^*+/−*^ and *Rd3*^*−/−*^*RetGC2*^*+/−*^ were bred to produce *Rd3*^*−/−*^*RetGC1*^*−/−*^ or *Rd3*^*−/−*^*RetGC2*^*−/−*^, and those were subsequently bred for the experiments described in Figure 1. Age-matched *Rd3*^*−/−*^ mice of the parental *rd3/rd3* strain were used for comparison. The animals were genotyped using PCR utilizing the respective genotype-specific primers and, in the case of *Rd3*^*−/−*^ background, DNA sequencing of the PCR fragments as described previously in full detail (24, 25, 31). The *R838S*^*+*^*RD3GFP*^*+*^ mice were produced by breeding heterozygous transgenic mouse line 932 (25) expressing human RD3GFP (*RD3GFP*^*+*^) to a heterozygous transgenic *R838S*^*+*^ mouse line 362 (16, 17) expressing the human R838S RetGC1. In the subsequent experiments, the *R838S*^*+*^*RD3GFP*^*+*^ mice were compared with their *R838S*^*+*^ littermates. Mice were fed the same diet and were housed in the same temperature- and humidity-controlled environment using 12 h/12 h light/dark cycle.

### RD3 expression and purification

Recombinant human RD3 was expressed from a pET11d vector in a BL21(DE3) Codon Plus *Escherichia coli* strain (Stratagene/Agilent Technologies) induced by isopropyl-β-d-thiogalactopyranoside, extracted from the inclusion bodies and purified by salt precipitation and dialysis as previously described (23, 24, 33). RD3^4ET^ was in addition purified using gel filtration on Sephacryl S-200 column.

### GCAP1 expression and purification

Myristoylated bovine GCAP1 for the *in vitro* assays was expressed from pET11d vector in a BLR(DE3) *E. coli* strain (both originated from Novagen/Calbiochem) harboring a pBB131 plasmid coding for a yeast N-myristoyl transferase and purified by calcium precipitation, butyl-Sepharose and Sephacryl S-100 chromatography using previously described procedures (43, 44, 45). The purity of GCAP1 preparations estimated by SDS gel electrophoresis was ≥90%.

### RetGC1 expression and guanylyl cyclase activity assays

Human recombinant RetGC1 was expressed from a modified Invitrogen pRCCMV vector in HEK293 cells transfected using calcium phosphate precipitation method, and the membrane fraction containing the expressed RetGC1 was isolated as previously described (15). The guanylyl cyclase activity was assayed as previously described in detail (15) with modification described (24). In brief, the assay mixture (25 μl) containing HEK293 membranes, 30 mM Mops–KOH (pH 7.2), 60 mM KCl, 4 mM NaCl, 1 mM DTT, 2 mM Ca^2+^/Mg^2+^/EGTA buffers, 0.9 mM free Mg^2+^, 0.3 mM ATP, 4 mM cGMP, 1 mM GTP, and 1 μCi of [α-^32^P]GTP, 100 μM zaprinast and dipyridamole, and 10 mM creatine phosphate/0.5 unit of creatine phosphokinase (Sigma–Aldrich) was incubated at 30 °C for 30 min, and the reaction was stopped by heat inactivation at 95° for 2 min. The resultant [^32^P]cGMP product was separated by TLC using fluorescently backed polyethyleneimine cellulose plates (Merck) developed in 0.2 M lithium chloride, cut from the plate, and eluted with 2 M lithium chloride in scintillation vials, and the radioactivity was counted using liquid scintillation. Mouse retinas for RetGC activity measurements were excised from the dark-adapted 3.5-week-old mice using infrared illumination (Kodak number 11 infrared filters) and a dissecting microscope fitted with an Excalibur infrared goggles, wrapped in aluminum foil, frozen in liquid nitrogen, and stored at −70 °C prior to their use in the cyclase activity assays, also conducted under infrared illumination. The incubation time for the reaction in that case was 12 min. The assay contained [^3^H]cGMP internal standard to ensure the lack of the cGMP product hydrolysis by retinal phosphodiesterase. Ca^2+^/EGTA buffers at 0.9 mM free Mg^2+^ were prepared using Tsien and Pozzan method (46) and verified by fluorescent indicator dyes as previously described in detail (44). Data fitting was performed using Synergy KaleidaGraph 4 software.

### ERG

Mice were dark-adapted overnight, their pupils were dilated by applying 1% tropicamide and 2.5% phenylephrine ophthalmic eye drops under dim red safelight illumination, and the mice were dark adapted for another 10 min. Full-field ERG in mice anesthetized by inhalation of 1.7 to 1.9% isoflurane (VEDCO)/air mix delivered by a Kent Scientific SomnoSuite setup at 50 ml/min was performed in the dark as previously described in detail (24, 25) using a Phoenix Research Laboratories Ganzfeld ERG2 instrument. A 505-nm 1-ms light pulse (∼1 × 10^6^ photoisomerizations/rod) was delivered through the infrared camera–guided corneal electrode/LED light source of the instrument.

### Retinal post-mortem morphology

Mice were anesthetized with a lethal dose of ketamine/xylazine injection, perfused through the heart with PBS, and then with 2.5% glutaraldehyde in PBS. The eyes were surgically removed and fixed overnight in 2.5% glutaraldehyde/2.5% formaldehyde in PBS (Electron Microscopy Sciences) at 4 °C. The fixed eyes were washed in PBS, soaked in PBS overnight, processed for paraffin embedding, sectioned at 5 μm, and stained with hematoxylin/eosin (AML Laboratories). The retinal sections were photographed using an Olympus BX21 microscope fitted with an Olympus MagnaFire camera, and then the identifiable photoreceptor nuclei per 100 μm in the ONL between the optic nerve and the periphery were counted and averaged from 425-μm frames.

### *In vivo* OCT

Mice were anesthetized using intraperitoneal injection of 20 mg/kg ketamine and 8 mg/kg xylazine. The pupils were dilated by applying 1% tropicamide and 2.5% phenylephrine ophthalmic eye drops ∼10 min before the scan. The B-scans of the retinas were acquired using an IIScience spectral domain OCT camera calibrated by the manufacturer at 2.47 μm/pixel axial scale and 3.5 μm/pixel lateral scale resolution and averaged from 200 to 400 frames. The thickness of the ONL layer was measured between the outer plexiform and the external limiting membrane reflective layers (47, 48). The OCT scans are presented with the choroid on top of the image and the ganglion cell layer on the bottom of the image.

### Antibodies

Rabbit RD3 polyclonal antibody 10929 was produced against purified recombinant mouse RD3 expressed in *E. coli* as described (24); anti-GCAP1 and anti-RetGC1 rabbit polyclonal (RRID: AB_2877058) antibodies were characterized previously (17, 18, 24, 49); rabbit polyclonal anti-GAPDH antibody was purchased from Invitrogen/Thermo Fisher Scientific (catalog no. PA1987). Polyclonal mouse antibody against CNG1 alpha subunit was produced in mice immunized by ∼22-kDa recombinant fragment, Ser^493^–Asp^684^, of a mouse CNG1α subunit expressed in *E. coli* from pET15b vector with a 6-His tag at the NH_2_ terminus and purified using affinity chromatography on a Ni column; for immunization, the antigen was injected subcutaneously, first using a complete Freund’s adjuvant and then incomplete Freund’s adjuvant (both from Thermo Fisher Scientific) for three subsequent booster injections delivered ca. 3 weeks apart. The blood was collected from mice anesthetized with a lethal injection of ketamine/xylazine and supplemented with 5 mM EDTA to prevent coagulation; the plasma was then separated from blood cells by centrifugation for 5 min at 5000*g,* 4 °C*,* and then twice for 10 min at 10,000*g*, 4 °C; the clear supernatant was aliquoted and frozen in liquid nitrogen and stored at −70 °C.

### Membrane binding assay

A 50-μl assay mixture containing HEK293 membranes in 30 mM Mops/KOH, pH 7.2, 60 mM KCl, 5 mM NaCl, 1 mM DTT, 0.1 mM ATP, 2 mM MgCl_2_, 0.4 mg/ml bovine serum albumin, 2 mM EGTA, inhibitors of proteases, 50 nM hRD3^4ET^, and various concentrations of L176F bGCAP1D6S was assembled on ice and then incubated for 15 min at room temperature. The assay mixture was loaded on top of 0.5 ml of cold 20% sucrose in 5 mM Tris, pH 7.5 in a centrifuge tube for a Beckman Optima TLS rotor, and centrifuged for 10 min at 40,000 rpm at 4 °C. First, the top 0.2 ml of supernatant was removed, then 0.4 ml of 5 mM Tris was carefully added on top of the remaining sucrose layer, and after that, all the supernatants were carefully aspirated, from top to bottom, to prevent nonspecific contamination of the pellet by RD3. The pellet was resuspended in 40 μl SDS-PAGE sample buffer, heated for 3 min at 100 °C and subjected to SDS-PAAG and Western immunoblotting using anti-RetGC1 and anti-RD3 antibodies.

### Immunoblotting

HEK293 membranes were subjected to Western immunoblotting after dissolving them in a Laemmli SDS sample buffer. The retinas from mice aged 3.5 weeks were excised in 20 μl of 10 mM Tris–HCl, pH 7.4, containing 1:100 dilution of a Millipore–Sigma protease inhibitor cocktail and homogenized for protein extraction in an Abcam radioimmunoprecipitation assay mixture of detergents containing the protease inhibitors, 300 μl/6 retinas. After 30 min of extraction on ice, the insoluble material was removed by centrifugation at 14,000*g* for 10 min, 4 °C, and the protein extract was mixed with equal volume of 2× Laemmli SDS sample buffer (Millipore–Sigma) and subjected to electrophoresis in linear or gradient polyacrylamide gels containing 0.1% SDS. Following the electrophoresis, the proteins were transferred overnight at 50 V constant voltage to Immobilon P membrane (Millipore) at 18 °C using Tris–glycine transfer buffer (Invitrogen/Thermo Fisher Scientific). The membranes were transiently stained by Ponceau S (Millipore–Sigma) dye solution in 1% acetic acid to mark the positions of molecular mass markers, destained by series of washes in water and Tris-buffered saline (Thermo Fisher Scientific) containing 0.5% Tween-20 (TTBS), blocked by SuperBlock (Thermo Fisher Scientific) solution in TTBS, probed by primary antibody for 1 h at room temperature, washed three times in TTBS and probed with secondary antibody for 1 h at room temperature. The secondary antibody was removed by washing three times for 15 min in TTBS and twice in Tris-buffered saline. The luminescence signal was developed using peroxidase-conjugated secondary polyclonal IgG (Cappel/MP Biomedical) and a Pierce SuperSignal Femto substrate kit (Thermo Fisher Scientific). The images were acquired and processed using a Fotodyne Luminous FX imager, ImageJ (National Institutes of Health) software; unmodified images were subjected to densitometry using Bio-Rad Multi-Analyst software.

### Confocal microscopy

Mice were euthanized by lethal injection of ketamine/xylazine and perfused with formaldehyde fixative solution, then the enucleated eyes were dissected using cryomicrotome, and the sections were mounted for microscopy as previously described (16, 25). The sections were washed three times in PBS containing 0.1 M glycine (pH 7.4), blocked for 1 h at 30 °C with the same solution containing 5% bovine serum albumin and 0.1% Triton X-100, incubated overnight at 4 °C and then 1 h at room temperature with the primary antibody, then washed with PBS solution three times for 15 min each, incubated with 1:400 diluted goat or donkey anti-rabbit and rabbit antimouse secondary antibody conjugated with AlexaFluor 568 or AlexaFluor 647 (Thermo Fisher Scientific), and washed four times for 15 min with PBS at room temperature. Confocal images were acquired using an Olympus FV1000 Spectral instrument controlled by FluoView FV10-ASW software, collecting the emitted fluorescence of different wavelengths in a sequential mode. Where indicated, the fluorescence was superimposed on a differential interference contrast image. The far-red fluorescence of AlexaFluor 647 in the images was assigned cyan pseudocolor. No changes were made to the original images, except for gamma correction applied to the whole image for better clarity in print.

### Statistics

Statistical significance of the differences was tested by ANOVA/Scheffe post hoc (confidence level 99%; alpha 0.01) multiple-pairs comparison or Student's *t* test (unpaired/unequal variance), using Synergy KaleidaGraph 4 software.

## Data availability

The data referred to in this article are contained within the article. Unprocessed data can be available from the corresponding author (adizhoor@salus.edu) upon reasonable request.

## Conflict of interest

The authors declare that they have no conflicts of interest with the contents of this article.
